# Brain Metabolic Correlates of Persistent Olfactory Dysfunction after SARS-Cov2 Infection

**DOI:** 10.3390/biomedicines9030287

**Published:** 2021-03-12

**Authors:** Maria Isabella Donegani, Alberto Miceli, Matteo Pardini, Matteo Bauckneht, Silvia Chiola, Michele Pennone, Cecilia Marini, Federico Massa, Stefano Raffa, Giulia Ferrarazzo, Dario Arnaldi, Gianmario Sambuceti, Flavio Nobili, Silvia Morbelli

**Affiliations:** 1 IRCCS Ospedale Policlinico San Martino, 16131 Genova, Italy; isabella.donegani@gmail.com (M.I.D.); albertomiceli23@gmail.com (A.M.); matteo.pardini@unige.it (M.P.); michele.pennone@hsanmartino.it (M.P.); cecilia.marini@unige.it (C.M.); fedemassa88@gmail.com (F.M.); Stefanoraffa@gmail.com (S.R.); giulia.ferrarazzo@gmail.com (G.F.); dario.arnaldi@unige.it (D.A.); sambuceti@unige.it (G.S.); flaviomariano.nobili@hsanmartino.it (F.N.); silviadaniela.morbelli@hsanmartino.it (S.M.); 2 Nuclear Medicine Unit, Department of Health Sciences, University of Genoa, 516126 Genoa, Italy; 3 Department of Neuroscience (DINOGMI), University of Genoa, 516126 Genoa, Italy; 4 Humanitas Clinical and Research Center–IRCCS, Via Manzoni 56, Rozzano, 20089 Milan, Italy; silvia.chiola@cancercenter.humanitas.it; 5 Department of Biomedical Sciences, Humanitas University, Via Rita Levi Montalcini 4, Pieve Emanuele, 20090 Milan, Italy; 6 CNR Institute of Molecular Bioimaging and Physiology (IBFM), 20090 Milano, Italy

**Keywords:** 18F-FDG PET, anosmia, COVID-19, SARS-CoV-2, olfactory dysfunction

## Abstract

We aimed to evaluate the brain hypometabolic signature of persistent isolated olfactory dysfunction after SARS-CoV-2 infection. Twenty-two patients underwent whole-body [^18^F]-FDG PET, including a dedicated brain acquisition at our institution between May and December 2020 following their recovery after SARS-Cov2 infection. Fourteen of these patients presented isolated persistent hyposmia (smell diskettes olfaction test was used). A voxel-wise analysis (using Statistical Parametric Mapping software version 8 (SPM8)) was performed to identify brain regions of relative hypometabolism in patients with hyposmia with respect to controls. Structural connectivity of these regions was assessed (BCB toolkit). Relative hypometabolism was demonstrated in bilateral parahippocampal and fusiform gyri and in left insula in patients with respect to controls. Structural connectivity maps highlighted the involvement of bilateral longitudinal fasciculi. This study provides evidence of cortical hypometabolism in patients with isolated persistent hyposmia after SARS-Cov2 infection. [^18^F]-FDG PET may play a role in the identification of long-term brain functional sequelae of COVID-19.

## 1. Introduction

Coronavirus disease 2019 (COVID-19) due to SARS-CoV-2 infection was initially thought to be mainly restricted to the respiratory system, but it has become evident that this disease also involves multiple other organs, including the central and peripheral nervous system. Indeed, neurological complications such as stroke, encephalopathy, delirium, meningitis, seizures, and cranial nerve deficits have been reported in patients with COVID-19 [1]. Besides these more severe manifestations and complications, other frequent symptoms of COVID-19 are loss of smell (anosmia) and taste (ageusia) which can occur as first symptoms of infection or in the absence of any other clinical features [1]. In a European study including more than 400 COVID-19 patients, olfactory dysfunction and ageusia were reported in 86% and 82% of patients, respectively [2]. Different underlying mechanisms have been advocated to explain the presence of anosmia in patients with COVID-19. These include olfactory cleft syndrome, direct damage of olfactory sensory neurons, postviral anosmia syndrome, cytokine storm, and/or impairment of the olfactory perception centers in the brain [3]. Indeed the olfactory bulb might represent a potential route of entry of SARS-CoV-2 in the CNS, and the investigation of the pathophysiology of olfactory dysfunction might help to further understand the pathogenesis and long-term implications of CNS involvement in COVID-19 [4]. In this framework, the availability of sensitive biomarkers tracking disease substrates might speed up the investigation of CNS involvement in patients with SARS-CoV-2 infection and might be used to monitor and better predict the risk of long-term effects. To date, there are few, partially conflicting, results on magnetic resonance imaging (MRI) abnormalities in patients with COVID-related anosmia [5,6]. Indeed normal, transiently increased, and even reduced volume of the olfactory bulb has been reported in patients with isolated or persistent anosmia [5,6]. Similarly, [^18^F]-Fluorodeoxyglucose ([^18^F]-FDG) PET data of COVID-19 patients with anosmia have to-date been made available only through case reports and small case series of patients with a self-reported reduction in smell [7,8,9,10,11]. This very preliminary evidence has been mainly acquired at the time of viral infection or just after recovery in patients affected by moderate to severe disease, thus complicating data interpretation [11].

[^18^F]-FDG PET may represent a sensitive tool to further confirm SARS-CoV-2 neurotropism through the olfactory pathway. Furthermore, given the potential functional and cognitive sequelae of COVID-19 and the established role of PET to support differential diagnosis of cognitive impairment, [^18^F]-FDG PET can represent a suitable tool to identify the concomitant involvement of cortical structures potentially relevant for subsequent persistent cognitive, sensory or emotion disturbances [8]. Moreover, an increasing number of patients showing persistent symptoms (such as fatigue, dyspnea, anosmia/dysgeusia, memory impairment, and pain) have been described after recovery from SARS-CoV-2, defining an emerging chronic syndrome, so-called Long Covid [12,13]. Given these premises, we aimed to evaluate the presence of regional brain hypometabolism in patients with persistent isolated and objectively-assessed olfactory dysfunction after recovery from SARS-CoV-2 infection.

## 2. Material and Methods

### 2.1. Patients

Patients with anosmia after SARS-CoV-2 infection were recruited among subjects who underwent whole-body [^18^F]-FDG PET, including a dedicated brain acquisition for clinical reasons other than SARS-CoV-2 infection in our institution between May and December 2020 (following their recovery after infection). The main inclusion criteria were previous SARS-CoV-2 infection, confirmed by polymerase chain reaction (PCR) at the time of initial symptoms, PET examinations performed during the recovery phase of SARS-CoV-2 infection, and an olfactory test still demonstrating olfactory dysfunction. The recovery phase was defined as when at least one negative swab test after infection was available. Exclusion criteria were demonstration of brain lesions on MRI, previous diagnosis of encephalopathy/encephalitis or cerebrovascular disorders due to or concomitant with the SARS-CoV-2 infection, or any other previous or current neurological or psychiatric disease. Patients that previously required mechanical ventilation or showed severe respiratory distress syndrome due to SARS-CoV-2 infection were also excluded, given the potential independent effect of these clinical scenarios on brain metabolism. Patients with a history of anosmia before SARS-CoV-2 infection, as well as those treated with chemotherapy in the last 3 months or previous radiotherapy in the head and neck district for oncological reasons, were also excluded. The study was approved by the Regional Ethical Committee (CER Liguria code 671/2020), all procedures and informed consent collection were in accordance with the ethical standards of the 1964 Helsinki declaration.

### 2.2. Olfactory Test

Olfaction was assessed by means of the Smell diskettes olfaction test [14] on the same day of PET examination. In fact, while self-reported newly onset loss of smell is important from an infection control perspective, self-reporting may result in misdiagnosis. The test was based on reusable diskettes as applicators of 8 different odorants. Using a questionnaire with illustrations, the test was designed as a triple forced multiple-choice test resulting in a score of 0 to 8 correct answers. Hyposmia was defined as making at least 2 mistakes on the questionnaire; the number of correct answers was recorded.

### 2.3. [^18^F]-FDG Brain PET Acquisition and Image Processing

A dedicated [^18^F]-FDG Brain PET acquisition was performed in all recruited patients according to the European Association of Nuclear Medicine (EANM) guidelines on two Siemens Biograph PET/CT systems (16 and mCT Flow 40, respectively) in the same center [15]. Images preprocessing was conducted using Statistical Parametric Mapping software version 8 (SPM8; Wellcome TrustCenter for Neuroimaging, London, UK) [16]. See Supplementary Materials for further details.

### 2.4. Voxel-Wise Analysis of Hypometabolic Signature of Olfactory Dysfunction after SARS-CoV-2 Infection

After preprocessing, smoothed images underwent a whole-brain voxel-wise group analysis to identify regions of relative hypometabolism with respect to a control group of 61 subjects consisting of 48 healthy controls acquired on the Biograph 16 system and previously recruited in our laboratory without any neurologic or psychiatric disease as detailed elsewhere [17] and thirteen subjects with smoldering multiple myeloma with both normal body and brain scans acquired on Biograph mCT Flow 40 PET/CT system (age 61.1 ± 11.1; 10 males). Patients with smoldering myeloma had no present or previous history of neurologic or psychiatric diseases and were never submitted to chemotherapy. Age, gender, and scanner type were included as nuisance variables in the analysis. We set a height threshold of family-wise error (FWE)-corrected *p* < 0.05 for multiple comparisons, at both the peak and cluster levels. Details on SPM analysis are included in the Supplementary Materials.

### 2.5. Structural Connectivity of Regions of Hypometabolism in Patients with Olfactory Dysfunction

The hypometabolic clusters in patients with hyposmia with respect to controls (hyposmia clusters) which had been obtained by means of the whole brain voxel-based analysis in SPM8, were saved as a volumetric region of interest (VOI). To assess the structural connectivity of metabolic correlates of hypo/anosmia after SARS-CoV-2 infection, we used the “Brain Connectivity and Behaviour” (BCB_ toolkit (18, http://www.toolkit.bcblab.com (accessed on 10 December 2020)), which included diffusion MRI data from healthy control subjects. Moreover, using the disconnectome pipeline in the BCB toolkit we computed structural connection maps of all voxels included in the hyposmia clusters by tracking fibers passing through them to identify their structural connectivity with other brain areas [18,19]. Briefly, the hypometabolic clusters present in patients with hyposmia with respect to controls (hyposmia clusters) and obtained by means of the whole brain voxel-based analysis in SPM8 were saved as VOI [16]. First, using the Tractotron pipeline, we evaluated the probability of the major white matter tracts crossing the hyposmia clusters and considered as significant only those voxels with a probability of at least 0.5. Moreover, using the disconnectome pipeline of the BCB toolkit [18,19], we computed the structural connection maps of all voxels included in the hyposmia clusters by tracking fibers passing through them to identify their structural connectivity with other brain areas as previously described [20]. Details about this procedure are detailed elsewhere [21].

## 3. Results

### 3.1. Patients

**Figure 1:**
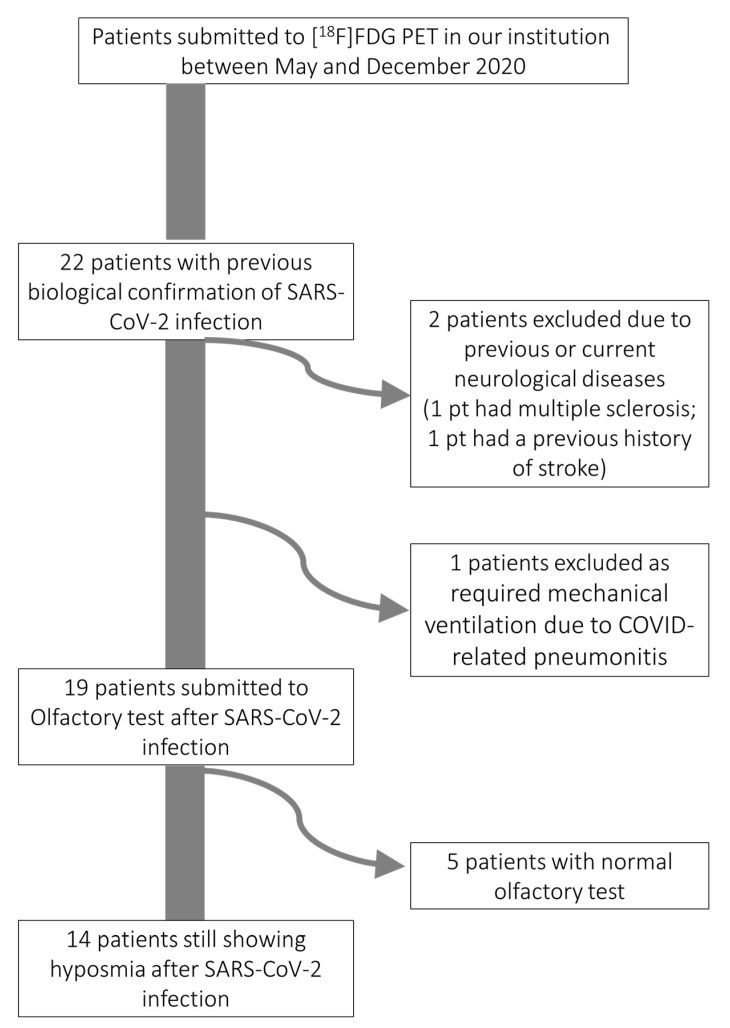
Flow-chart reporting steps that narrowed the final study group to fourteen patients still presenting with hyposmia during early recovery after SARS-CoV-2 infection. Pt, patients.

Twenty-two consecutive patients (12 males and 10 females; mean age 64 ± 10.5 years, range 35–79) in the recovery phase of SARS-CoV-2 infection were submitted to whole-body [^18^F]-FDG PET in our center from 1 May and 1 December 2020. [^18^F]-FDG PET was performed between 4 and 12 weeks after the first positive RT-PCR nasopharyngeal swab for SARS-CoV-2. Only nineteen of these patients met our inclusion criteria and were submitted to Smell diskettes olfaction test, which indicated the presence of hyposmia in fourteen of them who have been ultimately included in the present analyses. Figure 1 and its notes report the steps that narrowed the final study group. Further details on reasons for patients’ exclusion are reported in the Supplementary Materials. Characteristics of the 14 analyzed patients are detailed in Table 1.

### 3.2. Hypometabolism in Patients with Isolated Persistent Hyposmia after SARS-CoV-2 Infection

**Figure 2:**
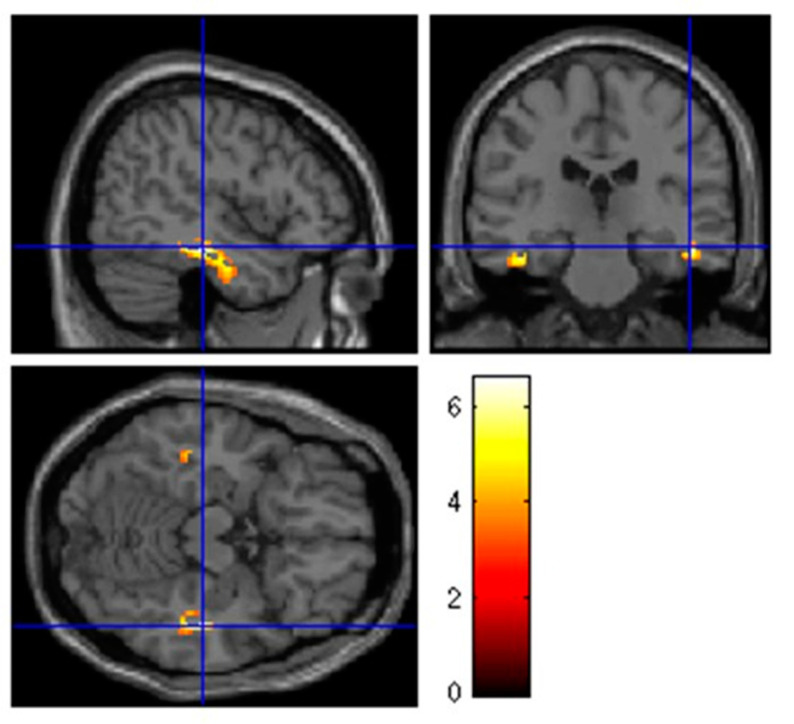
Hypometabolism with respect to controls in patients still presenting with hyposmia during early recovery after SARS-CoV-2 infection was highlighted in parahippocampal and fusiform gyri in both hemispheres (BA 20, 36, 37) and in the insula in the left hemisphere (BA 13). Height threshold of significance was set at *p* < 0.05 FWE-corrected at the cluster level. Regions of significant difference are shown color-graded in terms of Z values. Talairach coordinates and further details are available in Table 2.

With respect to the controls, patients with hyposmia after SARS-CoV-2 infection were characterized by relative hypometabolism in parahippocampal (Brodmann area (BA) 36), fusiform (BA 20 and 37) gyri in both hemispheres and in the insula in the left hemisphere (BA 13). Clusters of significant hypometabolism in patients with hyposmia after SARS-CoV-2 are reported in Figure 2. Details on coordinates and z-score are reported in Table 2.

### 3.3. Hyposmia Clusters Tractography and Connectivity

**Figure 3:**
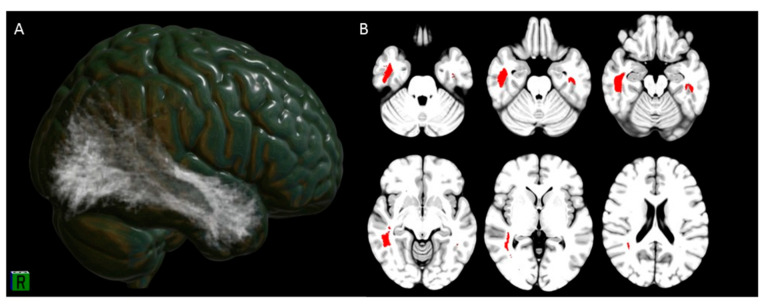
Structural connectivity of regions of hypometabolism in patients with olfactory dysfunction generated through the Brain Connectivity and Behaviour (BCB) toolkit (http://www.toolkit.bcblab.com (accessed on 10 December 2020)), which includes diffusion MRI data from healthy control subjects. Panel (**A**): The connectome map indicated a significant probability of connection of the hyposmia cluster with the inferior longitudinal fasciculus; Panel (**B**): tractography results of the hyposmia cluster.

The hyposmia cluster was found to be included in the bilateral longitudinal fasciculi (ILF) with a probability 0.82 and 1 for the left and right ILF, respectively. The tractography results for the hyposmia cluster are shown in Figure 3.

## 4. Discussion

The present brief communication provides a demonstration of brain hypometabolism, namely in the bilateral limbic cortex, in a group of patients with isolated persistent hyposmia proven by olfactory test more than four weeks after SARS-CoV-2 infection. The highlighted area of hypometabolism also encompassed the insula in the left hemisphere and included the bilateral ILF.

One of the ongoing hypotheses to explain the anosmia of patients with COVID-19 (in the absence of nasal congestion) is that the virus enters the CNS through the first neurons of the olfactory pathway located in the olfactory mucosa [1]. Post-infectious olfactory dysfunction is thought to be caused by damage to the olfactory epithelium or central olfactory processing pathways [22]. The present evidence of hypometabolism in two symmetric, similar regions within the limbic cortex may support the occurrence of a distal involvement of the olfactory pathway. Moreover, the bilateral involvement of key cortical structures is sound from the pathophysiological point of view. In fact, hyposmia might not be subjectively perceived in case of unilateral involvement of the olfactory pathway [23]. To-date one case report provided [^18^F]-FDG PET data in a patient with mild COVID-19 and isolated and persistent anosmia (in absence of any other COVID-related symptom) [10]. In fact, Karimi-Galougahi and colleagues reported the case of a 27-year-old woman with persistent anosmia for six weeks presenting hypometabolism of the left orbitofrontal cortex but with preserved metabolism in temporal cortex [10]. However, the evaluation of images was mainly based on visual inspection without observed-independent analysis or comparison with a control database which might have helped to more accurately evaluate a small region such as the medial temporal lobe. Evidence about the involvement of the limbic cortex after recovery from COVID-19 was also provided by two well-documented cases of patients submitted to whole body PET to assess metabolic activity of residual lung lesions just after COVID-related pneumonia [8]. Hypometabolism of the olfactory/rectus gyrus was present in both patients with additional hypometabolims within the amygdala, hippocampus, parahippocampus, cingulate cortex, pre-/post-central gyrus, thalamus/hypothalamus, cerebellum, pons, and the medulla in only one of them (who was not reporting anosmia). However, at the time of infection both these patients required hospitalization in intensive care unit and in one case, mechanical ventilation was needed. These more severe presentations and in particular mechanical ventilation may, at least in theory, have played a role on [^18^F]-FDG PET hypometabolic regions especially during early recovery [9,24,25]. However, this limitation does not apply to our patients’ group. Recently, Guedj and colleagues provided the first brain FDG PET data in Long COVID patients and again demonstrated bilateral hypometabolism in the bilateral rectal/orbital gyrus, amygdala and the hippocampus, brainstem and bilateral cerebellum. Thus, our findings largely confirm the topography of brain hypometabolism in patients in long COVID patients although associated with persistent hyposmia or with other persistent functional complaints [26]. The presence and the topography of hypometabolism after recovery in all the mentioned case reports, in previous small group studies and in our hyposmic group repetitively highlighted an involvement of limbic regions and might point to the risk of developing long-term neurological (possibly cognitive) sequelae, a hypothesis requiring studies in patients with a much longer recovery from infection [8,27,28]. Indeed, olfactory cortical area feeds into multimodal integration relevant for cognition control and the hippocampal regions is known to exchange input for storage of olfactory memory (also relevant for working memory [29]). Almeria et al. evaluated the impact of COVID-19 on neurocognitive performance in thirty-five patients with confirmed COVID-19 infection and found that the presence of anosmia and dysgeusia at the time of infection were among the main risk factors for cognitive impairment related with attention, memory and executive function [28].

Of note, the present group of patients showed hypometabolism also in the insula in the left hemisphere. The insula is densely interconnected with orbitofrontal and anterior cingulate cortices, amygdala, and hippocampus [30] and plays a key role in processing self-awareness. Indeed, olfaction aims to provide critical information about the environment subsequently directed at the cortical level also for multisensory integration.

Regarding the connectivity data, the involvement of the ILF is in line with observations of its role in hyposmia in Parkinson’s Disease [31]. Interestingly, the ILF has been shown to be affected early on in viral infections, such as in HIV [32] and hepatitis C virus (HCV) [33].

Finally, it should be noted that MRI cortical signal has been evaluated in patients with COVID-19 and anosmia in few small studies. However, abnormalities have been substantially reported in the very early phase of infection [5,6,34,35]. In this framework, while the presence of hypometabolism at [^18^F]-FDG PET cannot prove the direct spread of the virus along the olfactory pathway and cortex, the high sensitivity of FDG PET for cortical deafferentation may act as a measurable biomarker of persistent impairment of the transmission along the olfactory pathway [36]. Further investigation might help to understand if [^18^F]-FDG PET data could be used to predict the prognosis of olfactory function recovery also at the single patient level [34]. The present study has some limitations, mainly related to its naturalistic observational nature and to the small group of patients being submitted to [^18^F]-FDG PET for other clinical reasons, including the suspect or follow-up of oncological diseases. Brain lesions were radiologically excluded in all patients, and to reduce the potential confounding effect of comorbidities, we also excluded patients submitted to chemotherapy in the last three months or who underwent radiotherapy in the head and neck district. Indeed despite the small number of included patients, the present study provides a group analysis on brain metabolism of patients with persisting olfactory dysfunction after SARS-CoV-2 infection for the first time proven by olfactory test. The demonstration of hyposmia by means of an olfactory test (that was not possible in larger epidemiologic studies) and the exclusion of patients who suffered from COVID-related pneumonia, requested COVID-oriented treatment or mechanic ventilation is a further strength of the present study [37].

## 5. Conclusions

The COVID-19 outbreak has impacted clinical neurology in previous months, and other challenges might come in the next future. For several reasons, a not negligible number of neurological and cognitive complaints might emerge once the acute phase of the pandemic crisis is overcome. It will be of great scientific and clinical relevance to describe COVID-19 related cognitive symptoms (likely to be reversible or in any case not progressive) and to identify and characterize biomarkers that will help us to support clinical differential diagnosis with respect to cognitive impairment caused by neurodegenerative disease. [^18^F]-FDG PET might play a role in this clinical setting. To this aim, we will need to be aware of the confounding effect of subtle sequelae of SARS-COV2 infection and on their reflection on PET and other biomarkers as demonstrated in the present study.

## Figures and Tables

**Table 1:** Patients’ Characteristics.

Characteristics	SARS-CoV-2 Patients with Hyposmia (*n* = 14) *
Age (years)	64.4 ± 10.9 (range 51–79)
Sex	
Male	7/14
Female	7/14
Time Since Diagnosis of SARS-CoV-2 infection (weeks) †	8.3 ± 2.1 (range 4–14)
Time Since first negative swab after proven SARS-CoV-2 infection (weeks)	4.0 ± 1.9 (range 1–7)
*Olfactory test* (number of correct answers)	
6/8	2
5/8	2
4/8	5
3/8	2
2/8	3

Values are shown as mean ± standard deviation (range). * None of these patients was complaining of other known possible sequelae of COVID-19 such as fatigue, chest pain, dyspnea, or reported any other focal neurological signs both at the time of SARS-CoV-2 infection and at the time of PET. † None of the patients had proven previous COVID-related lung involvement or previously received steroids, hydroxychloroquine, or other medication specifically aimed to support patients’ response to COVID-19 (other than paracetamol).

**Table 2:** Whole-brain mapping of relative hypometabolism in patients with persistent olfactory dysfunction after SARS-CoV-2 infection with respect to controls.

	Cluster Level		Peak Level					
Cluster Extent	Corrected *p*-Value	Cortical Region	Maximum Zscore		Talairach Coordinates		Cortical Region	BA
260	0.032							
		R-limbic	5.68	45	−26	−9	Parahippocampal Gyrus	36
		R-Temporal	3.45	45	−22	−11	Fusiform Gyrus	20
		R-Temporal	3.41	45	−33	−9	Fusiform Gyrus	37
155	0.034	L-Limbic	5.15	−39	−29	−13	Parahippocampal Gyrus	36
		L-sublobar	3.36	−44	−37	17	Insula	13

*p* < 0.05, corrected for multiple comparisons with the Family-Wise error option both at peak and cluster level were accepted as statistically significant. In the ‘cluster level’ section on the left, the corrected *p*-value and the brain lobe with hypometabolism are reported. In the ‘peak level’ section on the right, the Z score and peak coordinates, the corresponding cortical region, and Brodmann area (BA) are reported. L, left; R, right.

## Data Availability

The datasets used and/or analyzed during the current study are available from the corresponding author on reasonable request.

## Supplementary Materials

Descriptive Details on patients’ selection and further details on image preprocessing and analysis are available online at https://www.mdpi.com/2227-9059/9/3/287/s1.
