# Supplementary material for: Brain Metabolic Correlates of Persistent Olfactory Dysfunction after SARS-Cov2 Infection
Source: Biomedicines. 2021 Mar 12;9(3):287. doi: 10.3390/biomedicines9030287 (PMC7998481; doi:10.3390/biomedicines9030287)
Supplement: Supplementary file 1 [file biomedicines-09-00287-s001.pdf]

## Supplementary Materials.

### Descriptive Details on patients' selection

Twenty-two consecutive patients (12 males and 10 females; mean age 64 ± 10.5 years, range 35-79) were submitted to whole body 18F-FDG PET in our center from May the 1st and December the 1st 2020 after their recovery from SARS-CoV-2.

Clinical indication for 18F-FDG PET included diagnosis (i.e. characterization lung nodules) and follow up of oncologic diseases (Hodking's and Non Hodking's Lymhoma, breast cancer, melanoma and multiple myeloma). 18F-FDG PET was performed between 4 and 12 weeks after first positive RT-PCR nasofaringeal swab for SARS-CoV-2. Three patients were excluded as they were not meeting our inclusion criteria (one patients had history of stroke, one of multiple sclerosis and one patient required mechanical ventilation due to COVID-related pneumonitis). The remaining nineteen patients were submitted to Smell diskettes olfaction test which indicated the presence of hyposmia in fourteen of them.

Only brain 18F-FDG PET scans of the subgroup of fourteen patients with objectively-proven persistent hyposmia were used in the following SPM analyses. Characteristics of the 14 analyzed patients are detailed in Table 1.

Of note, none of the included patients was complaining of other known possible sequelae of COVID-19 such as fatigue, chest pain, dyspnea or reported any other focal neurological signs both at the time of SARS-CoV-2 infection and at the time of PET. Moreover none of them had proven previous COVID-related lung involvement or previously received steroids,

hydroxychloroquine or other medication specifically aimed to support patients' response to COVID-19 (other than paracetamol).

## Further Details on Images preprocessing and analysis

### 18F-FDG Brain PET acquisition and Image processing

FDG PET was performed according to the European Association of Nuclear Medicine (EANM) guidelines on two Siemens Biograph PET/CT systems (16 and mCT Flow 40) in the same center (Varrone et al 2009). Briefly, subjects fasted for at least 6 hours. Before radiopharmaceutical injection, blood glucose was checked and was <140 mg/dl in all subjects. 200-250 MBq of 18F-FDG were injected via a venous cannula after a 15-minute rest in a silent and dimly light room, with unplugged ears and closed eyes. PET scan started approximately 45 minutes after the injection, lasting 12 minutes. The acquired images were reconstructed with an ordered subset-expectation maximization algorithm following the standard protocols used for clinical purposes and embedded in the equipment workstations. Attenuation correction was based on computed tomography scan.

Image preprocessing was conducted using the MATLAB and Statistical Parametric Mapping software (SPM8; Wellcome TrustCenter for Neuroimaging, London, UK) (Friston *et al.*, 1994) . Brain FDG PET images were then subjected to affine and nonlinear spatial normalization into Talairach and Tournoux space using SPM8. All the default choices of SPM were followed with the exception of spatial normalization. For this study, the H215O SPM-default template was replaced by a brain FDG PET as detailed elsewhere (Morbelli *et al.*, 2010). The spatially

normalized set of images was then smoothed with a 8-mm isotropic Gaussian filter to blur individual variations in gyral anatomy and to increase the signal-to-noise ratio.

**Voxel-wise analysis of hypometabolic signature of olfactory dysfunction after SARS-CoV-2 infection**

After preprocessing, smoothed images underwent a whole-brain voxel wise group analysis to identify regions of relative hypometabolism with respect to a control group of 61 subjects consisting of 48 healthy controls acquired on the Biograph 16 system and previously recruited in our laboratory (Morbelli *et al.*, 2017) and thirteen subjects with smoldering multiple myeloma with both normal body and brain scans acquired on Biograph mCT Flow 40 PET/CT system and without brain lesions on MRI. In all the analyses, the standard 0.8 gray matter threshold masking and the default value of 50 for the grand mean scaling were used. We set a height threshold of family-wise error (FWE)-corrected $p<0.05$ for multiple comparisons both peak and cluster level. Only cluster containing at least 50 voxels were considered. Age gender and scanner were included as nuisance variables in the analysis.

**Structural connectivity of regions of hypometabolism in patients with olfactory dysfunction**

To assess the structural connectivity of metabolic correlates of hypo/anosmia after SARS-CoV-2 infection, we used the BCB toolkit (Foulon et al., 2018, http://www.toolkit.bcblab.com), which includes diffusion MRI data from healthy control subjects (Rojkova et al.,2016). Details about this procedure are detailed elsewhere (Massa *et al.*, 2020) and here only summarized. Briefly the hypometabolic clusters present in patients with hyposmia with respect to controls (hyposmia clusters) and obtained by means of the whole brain voxel based analysis in SPM8 were saved as

VOI. First, using the Tractotron pipeline, we evaluated the probability of the major white matter tracts to cross the hyposmia clusters and considered as significant only those voxels with a probability of at least 0.5. Moreover, using the disconnectome pipeline of the BCB toolkit (Foulon et al., 2018), we computed the structural connection maps of all voxels included in the hyposmia clusters by tracking fibers passing through them to identify its structural connectivity with other brain area as previously described (Thiebaut de Schotten et al., 2011).

References

Friston KJ, Holmes AP, Worsley KJ, Poline JP, Frith CD, Frackowiak RS. Statistical parametric maps in functional imaging: a general linear approach. Human Brain Mapping. 1994; 2:189–210.

Massa F, Grisanti S, Brugnolo A, Doglione E, Orso B, Morbelli S et al. The role of anterior prefrontal cortex in prospective memory: an exploratory FDG-PET study in early Alzheimer's disease. Neurobiol Aging. 2020;96:117-127.

Morbelli S, Piccardo A, Villavecchia G, Dessi B, Brugnolo A, Piccini A, Caroli A, Frisoni G, Rodriguez G, Nobili F. Mapping brain morphological and functional conversion patterns in amnestic MCI: a voxel-based MRI and FDG-PET study. Eur J Nucl Med Mol Imaging. 2010; 37:36-45.

Morbelli S, Bauckneht M, Arnaldi D, Picco A, Pardini M, Brugnolo A et al. 18F-FDG PET diagnostic and prognostic patterns do not overlap in Alzheimer's disease (AD) patients at the mild cognitive impairment (MCI) stage. Eur J Nucl Med Mol Imaging. 2017;44:2073-2083.

Rojkova K, Volle E, Urbanski M, Humbert F, Dell'Acqua F, Thiebaut de Schotten M. Atlasing the frontal lobe connections and their variability due to age and education: a spherical deconvolution tractography study. Brain Struct Funct. 2016;221:1751-66.

Thiebaut de Schotten M, Dell'Acqua F, Ratiu P, Leslie A, Howells H, Cabanis E, Iba-Zizen MT, Plaisant O, Simmons A, Dronkers NF, Corkin S, Catani M. From Phineas Gage and Monsieur Leborgne to H.M.: Revisiting Disconnection Syndromes. Cereb Cortex. 2015;25:4812-27.

Varrone A, Asenbaum S, Vander Borght T, Booij J, Nobili F, Någren K, Darcourt J, Kapucu OL, Tatsch K, Bartenstein P, Van Laere K; European Association of Nuclear Medicine Neuroimaging Committee. EANM procedure guidelines for PET brain imaging using [18F]FDG, version 2. Eur J Nucl Med Mol Imaging. 2009 Dec;36:2103-10
